# Machine learning and deep learning applied to EEG and fNIRS for early autism spectrum disorder diagnosis: a systematic review

**DOI:** 10.3389/fpsyt.2026.1668914

**Published:** 2026-02-03

**Authors:** Andrea De Giacomo, Roberta Palmieri, Emanuele Francesco Russo, Ilaria Pizzolorusso, Roberta Brandi, Francesca Magistro, Michele Giuseppe Di Cesare, Sara Quattrocelli, Daniela Cardone, David Perpetuini, Arcangelo Merla

**Affiliations:** 1 Department of Translational Biomedicine and Neurosciences (DiBraiN), University Hospital, Bari, Italy; 2 Child and Adolescent Neuropsychiatry Unit, Department of Mental Health, ASL Local Health Authority (ASL), Foggia, Italy; 3 Padre Pio Foundation and Rehabilitation Centers, San Giovanni Rotondo, Foggia, Italy; 4 Department of Engineering and Geology, University “G. d’Annunzio” of Chieti-Pescara, Pescara, Italy

**Keywords:** autism spectrum disorder, deep learning, EEG, fNIRS, machine learning, neuroimaging

## Abstract

**Introduction:**

Autism Spectrum Disorder (ASD) presents considerable diagnostic challenges due to its heterogeneous nature and early developmental onset. In recent years, the convergence of noninvasive neuroimaging modalities such as Electroencephalography (EEG) and Functional Near Infrared Spectroscopy (fNIRS) with machine learning (ML) and deep learning (DL) techniques has opened new avenues for uncovering objective biomarkers of ASD. EEG offers millisecond level resolution of brain electrical activity, while fNIRS tracks hemodynamic responses tied to neuronal function, making the two methods complementary. This review aims to investigate the state of the art of the applications of EEG and fNIRS to ASD patients combined with ML and DL approaches.

**Methods:**

To this goal, Scopus and PubMed databases were searched, and following the PRISMA guidelines, 27 peer reviewed studies published between 2019 and 2024 were included in the survey.

**Results:**

The results showed consistent patterns across the studies, including alterations in neural oscillations and disruptions in connectivity within key brain regions related to social communication and cognition. However, a strong heterogeneity was assessed regarding probes montages, preprocessing workflows, and classification models employed, limiting the feasibility of a metanalysis.

**Discussion:**

The results demonstrated the potential of DL and ML algorithms applied to EEG and fNIRS signals for early ASD assessment, supporting the development of personalized intervention strategies grounded in robust neurophysiological evidence.

## 1 Introduction

Autism Spectrum Disorder (ASD) is a complex neurodevelopmental condition characterized by challenges in social communication and the presence of restricted and repetitive behaviors (1). Early identification of ASD is critical, as timely intervention can significantly improve developmental outcomes (2). However, the heterogeneity of ASD presents significant challenges in establishing reliable and objective diagnostic methods (3). Non-invasive neuroimaging techniques, such as Electroencephalography (EEG) and Functional Near-Infrared Spectroscopy (fNIRS), have emerged as promising tools for studying the neural underpinnings of ASD. These modalities offer complementary insights into brain function, with the potential to uncover neurophysiological biomarkers that could revolutionize early diagnosis and intervention strategies. Both EEG and fNIRS, in fact, provide critical insights into brain activity from distinct perspectives. While fNIRS measures hemodynamic responses associated with neuronal activity, EEG records the brain’s electrical activity directly from the scalp. The integration of these methods offers a comprehensive view of the brain’s dynamic interplay between electrophysiological and hemodynamic signals, a synergy that could be particularly valuable in the study of neurodevelopmental disorders such as ASD, where identifying specific biomarkers is essential for early diagnosis and intervention.

In detail, EEG measures the brain’s electrical activity through electrodes placed on the scalp. It records the alternating electrical signals generated by synchronized neuronal activity, particularly the currents produced during synaptic excitations. While EEG provides exceptional temporal resolution, capturing neural dynamics on a millisecond timescale, its spatial resolution is limited compared to techniques like functional magnetic resonance imaging (fMRI). Nonetheless, EEG excels in detecting abnormal electrical patterns, which makes it a cornerstone of clinical neurology for diagnosing epilepsy, monitoring brain injuries, and assessing brain states such as alertness, coma, or sleep (4–6). Furthermore, EEG enables the study of event-related potentials (ERPs), which are voltage changes linked to specific sensory, motor, or cognitive events, making it particularly valuable in studying perception, attention, memory, and language processing (7). The non-invasive nature and ease of use of EEG make it especially suitable for early diagnosis in pediatric populations also providing a valuable tool for monitoring the effectiveness of therapeutic interventions and supporting personalized treatment approaches (8).

Similarly, fNIRS is a non-invasive modality that uses near-infrared light to assess variations in hemoglobin oxygenation and cerebral hemodynamics, which are directly related to neuronal activity. Due to its safety, portability, and real-time capabilities, fNIRS is especially advantageous for investigating cerebral development in pediatric populations, enabling studies of brain function during both social interactions and cognitive tasks (9, 10). The technique works by transmitting near-infrared photons through biological tissues, where their passage is modulated by reflection, scattering, and absorption. By focusing on specific chromophores such as oxygenated hemoglobin (HbO2) and deoxygenated hemoglobin (HbR), fNIRS offers valuable insights into cerebral oxygenation and blood flow, making it a powerful tool for both research and clinical settings (11).

In the field of ASD detection, fNIRS, although limited in spatial resolution to superficial cortical structures, offers a non-invasive means of assessing brain activity in pediatric populations. Its ability to monitor brain function during social interactions and cognitive tasks provides significant advantages for investigating the neurodevelopmental trajectory of children, including those with ASD. On the other hand, EEG provides a high temporal resolution, which is crucial for studying the rapid cognitive processes involved in ASD, with studies that have identified abnormalities in cerebral connectivity that may underpin the sensory integration, attention, and social communication impairments characteristic of ASD (12).

Moreover, both EEG and fNIRS are particularly valuable in the context of early childhood, as this developmental stage is marked by heightened neuroplasticity (13). The brain’s ability to adapt and reorganize in response to environmental stimuli and therapeutic interventions is at its peak during this period, making early intervention crucial for shaping cognitive, social, and emotional functions. Early detection of abnormal cerebral signals, facilitated by both EEG and fNIRS, allows for the implementation of targeted interventions that can have a lasting, positive impact on a child’s neurocognitive trajectory (12, 14). One of the key advantages of both EEG and fNIRS is their practical applicability in pediatric research. In fact, both EEG and fNIRS are non-invasive, portable, and safe, making them particularly well-suited for use with infants. Their lightweight and adaptable design enables data collection in natural environments, such as homes or classrooms, reducing the need for children to undergo potentially stressful clinical procedures. Furthermore, the silent operation of both techniques eliminates discomfort associated with the noise produced by other imaging modalities, enhancing their acceptability among young children. These practical advantages, coupled with their ability to capture real-time brain activity, make EEG and fNIRS ideal tools for the early monitoring and diagnosis of neurodevelopmental disorders, facilitating the adoption of early intervention strategies.

In parallel with advancements in neuroimaging, machine learning (ML) is increasingly being applied to enhance the accuracy and reliability of medical diagnostics. By analyzing large, complex datasets, those algorithms can identify patterns that may be missed by traditional methods, improving the precision of diagnoses. Deep learning (DL) techniques have shown great promise in autonomously learning from data, leading to more personalized and timely diagnoses. This has particular relevance for the early diagnosis of ASD, since ML and DL algorithms can aid in detecting abnormal cerebral signals and patterns, and improve diagnostic accuracy (15).

The aim of this review is to provide a comprehensive overview of the application of neuroimaging techniques, particularly EEG and fNIRS, combined with ML and DL algorithms, in the diagnosis of ASD. This review will explore the identification of neurophysiological biomarkers, the involvement of key brain regions, and the potential of these technologies to enable early diagnosis and personalized interventions. Key findings, current limitations, and future perspectives in this field will be critically discussed.

## 2 Materials and methods

### 2.1 Search strategy

The systematic review was conducted following the Preferred Reporting Items for Systematic Reviews and Meta-Analyses (PRISMA) guidelines (16) and the Joanna Briggs Institute (JBI) critical appraisal tools (17). The databases employed for the search were PubMed and Scopus. The final search was carried out on 15 February 2025. The search was performed using an algorithm that combined terms related to the identification of neurophysiological biomarkers in ASD using EEG and/or fNIRS in conjunction with ML and DL techniques. The search terms employed were based on the following two primary queries, which are detailed in **Table 1**.

**Table 1:** Search queries and filters used for PubMed and Scopus in the systematic review.

Database	Search terms	Filters applied
PubMed	(“Autism Spectrum Disorder” OR ASD) AND (EEG OR electroencephalography) AND (“machine learning” OR “deep learning”) AND (classification OR diagnosis) (“Autism Spectrum Disorder” OR ASD) AND (fNIRS OR “functional near infrared spectroscopy”) AND (“machine learning” OR “deep learning”) AND (“biomarker” OR “signal analysis”)	“Article” and “Clinical trial” (searched by topic), excluding review articles
Scopus	(“Autism Spectrum Disorder” OR ASD) AND (EEG OR electroencephalography) AND (“machine learning” OR “deep learning”) AND (classification OR diagnosis) (“Autism Spectrum Disorder” OR ASD) AND (fNIRS OR “functional near infrared spectroscopy”) AND (“machine learning” OR “deep learning”) AND (“biomarker” OR “signal analysis”)	“Articles” (searched through title/abstract/keywords), excluding review articles

In PubMed, these search terms were applied by topic using the filters “Article” and “Clinical trial” while explicitly excluding review articles, whereas in Scopus the search terms were employed in the title, abstract, and keywords fields using the “Articles” filter with a similar exclusion of reviews. No additional restrictions on the date of publication were applied beyond the time window of 2019 to 2024, in order to provide a description of the major methodological advances in this field.

### 2.2 Selection criteria

For inclusion in this systematic review, studies must:

- Peer-reviewed articles published between 2019 and 2024.
- Focus on the application of EEG and/or fNIRS techniques to identify neurophysiological biomarkers in ASD.
- Incorporate ML or DL methodologies for data analysis and classification.
- Conducted on ASD subjects.

Studies were excluded if they did not meet the inclusion criteria or if they were classified as review articles, brief communications, notes, theoretical articles, letters to the editor, or unpublished works that had not undergone peer review. Specifically, studies were excluded if they lacked sufficient methodological detail regarding data acquisition, preprocessing, or analysis (Reason 1), or if they did not present original empirical data (Reason 2). Regarding Reason 1, information concerning the helmet montage (number of measurements points and coverage), the characteristics of the filters (type of filter, cut-off frequencies), the methods employed for the artifact reduction, and the ML/DL approach (composition of the dataset, description of the machineries, cross-validation, and testing approaches) should have been clearly stated for the inclusion in the review. Papers written in a language other than English were excluded, as well.

**Figure 1:**
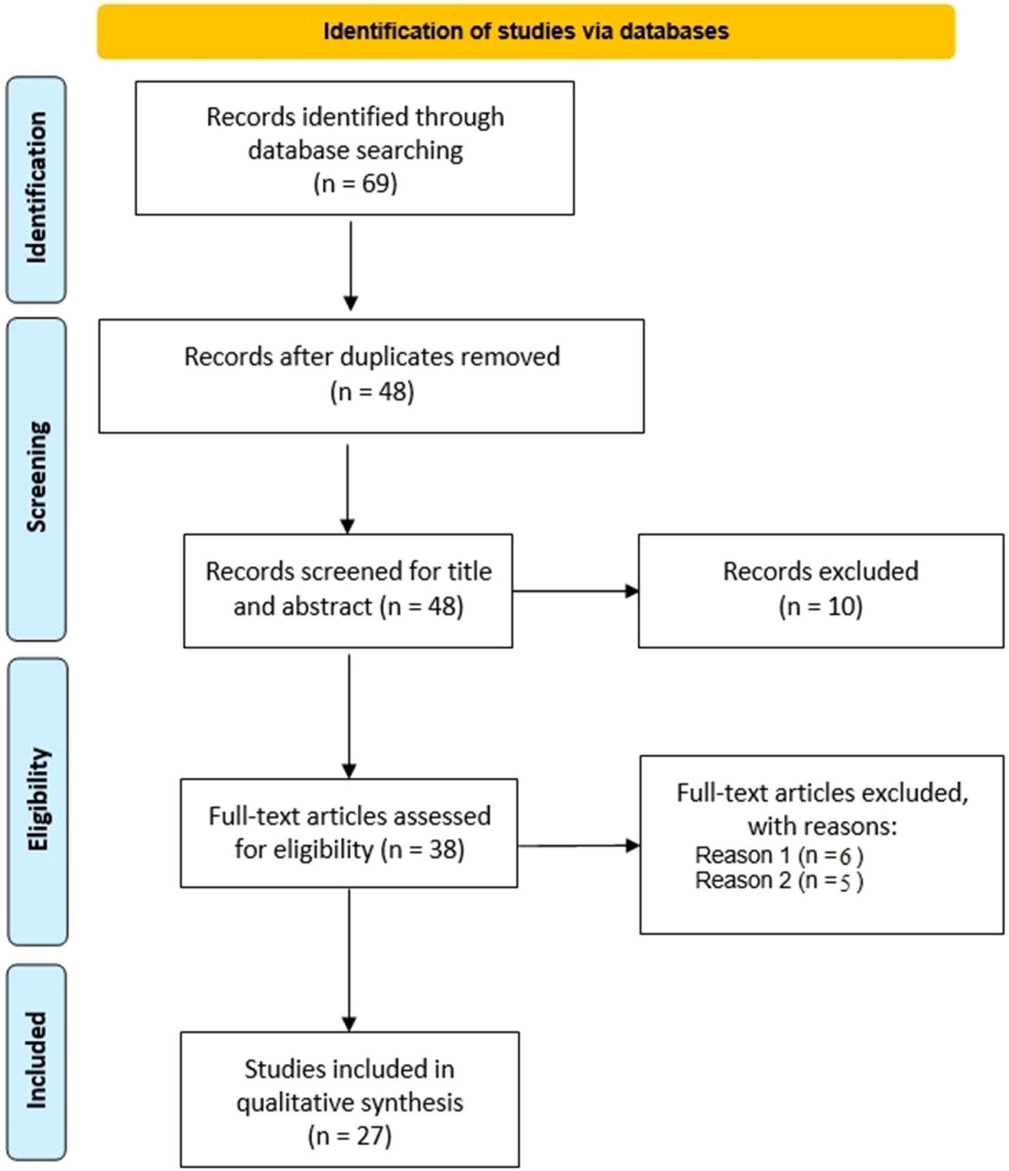
PRISMA 2020 flow diagram for new systematic reviews that included searches of databases only.

**Figure 1** shows a flowchart of the study selection process. Initially, a total of 69 articles were identified (including duplicates) across PubMed and Scopus.

After the duplicates were eliminated, 48 unique articles remained, the titles and abstracts of all potentially pertinent papers were independently reviewed by two reviewers (EFR and DP) for inclusion. In case of disagreement, the judgment of a third reviewer led to a consensus. The same two reviewers then retrieved full-text studies and separately checked them for inclusion. If an agreement could not be reached through conversation, a third reviewer’s judgment was used to settle disputes (MGDC), 27 articles were finally included in the review.

The following information was taken out: title, authors, year of publication, signal employed, features employed, processing techniques, ML or DL model used, main findings, implications.

To quantify how consistently neurophysiological findings and preprocessing strategies are reported across the included studies, an inter-study agreement analysis was performed on the variables associated with the metrics employed for neuroimaging data analysis and on the preprocessing techniques. Each study was treated as an independent “rater” and each variable as an “item”. Variables were coded as binary: 1 = explicitly reported, 0 = not reported. For each variable, the prevalence (% of studies reporting it), observed agreement $P_{o}$ across studies, and the Prevalence-Adjusted Bias-Adjusted Kappa (PABAK, computed as $2P_{o}-1$) to mitigate inflation of raw agreement in low-prevalence variables. Moreover, a rigorous variable-wise agreement analysis using the Fleiss’ Kappa and Krippendorff’s Alpha was performed.

## 3 Results

A total of 27 studies published between 2019 and 2024 were included in this review, focusing on the identification of neurophysiological markers, signal preprocessing methods, feature extraction techniques, and classification models. Key findings include the adoption of standard preprocessing pipelines to mitigate artifacts and improve signal quality, the application of advanced ML and DL algorithms that achieved promising diagnostic accuracy, and detailed insights into limitations and future directions. The results are organized into three sections: Neurophysiological Markers and Brain Regions Implicated in ASD; Signal Preprocessing Techniques; Classification Algorithms and Models Employed. This structure emphasizes how these three components interact to distinguish studies from one another and how their integration could lead to novel insights and improved results. Collectively, the review supports the potential of EEG- and fNIRS-based ML systems to advance ASD diagnostics.

### 3.1 Neurophysiological markers and brain regions implicated in ASD

To better understand the abnormalities caused by ASD in children, most EEG studies focused on neurophysiological alterations in the alpha, beta, and theta bands. These studies consistently highlighted significant differences in brain activity patterns between children with ASD and typically developing (TD) controls. For instance, Bakheet et al. reported increased theta power in the frontal cortex, suggesting impairments in cognitive processing, attention, and memory functions (18). Similarly, Martinez et al. observed heightened theta and alpha power in the frontal regions, indicating dysfunctions in attention regulation and inhibitory processes (19). Conversely, reduced alpha power was often reported in the occipital and frontal areas, potentially explaining difficulties in attention focus and inhibition. This inability to modulate alpha activity may interfere with the processing and response to social stimuli, contributing to the social and emotional challenges observed in ASD (20, 21). Beta band connectivity also revealed abnormalities, with decreased synchronization between frontal and temporal regions, as noted by Wang et al., who suggested that these deficits could reflect impaired inter-regional communication critical for social and sensory processing (22). Rogala et al. further explored these findings, reporting reduced beta wave synchrony and altered global connectivity, particularly in the alpha and beta bands, reinforcing the hypothesis of disrupted inter-regional communication in ASD (23).

fNIRS studies, on the other hand, focused on alterations in regions associated with language and social cognition, such as the inferior frontal gyrus (IFG) and temporal cortex (TC). Xu et al. observed non-stationary temporal dynamics in these regions, potentially serving as markers for ASD classification (24). Zhu et al. identified enhanced regional connectivity in the left inferior frontal gyrus (LIFG), highlighting its central role in the neural disruptions linked to ASD (25). These findings align with commonly observed language deficits in ASD, involving regions such as Broca’s area, the superior temporal gyrus (STG), and the left temporal lobe (LTL). The latter, critical for language comprehension and auditory memory, was found to exhibit significant alterations in ASD, impacting cognitive functions related to semantic processing and auditory information integration (26, 27).

Interestingly, both EEG and fNIRS studies introduced promising novel markers, such as entropy-based measures, capable of distinguishing children with ASD from TD controls. Additionally, neurophysiological improvements following therapeutic interventions were observed. Techniques like mindfulness were shown to enhance theta and alpha activity, suggesting that EEG and fNIRS could serve not only as diagnostic tools but also as methods for monitoring treatment efficacy, particularly in attention regulation and emotional control (28, 29). The main results of the studies investigated in this section are reported in **Table 2**.

**Table 2:** Summary of key EEG and fNIRS findings in children with ASD, including altered spectral patterns, connectivity changes, and neurophysiological markers associated with cognitive and social impairments.

Authors	Signal employed	Channels montage	Findings	Implications
Bakheet et al. (18)	EEG	8 channels located in the Fusiform Gyrus.	Increased theta power in frontal cortex	Cognitive processing, attention, and memory impairments
Martinez et al. (19)	EEG	7 Channels (F3-M2, F4-M1, C3-M2, C4-M1, O1-M2, O2-M1, Cz–O1)	Heightened theta and alpha in frontal regions	Attention regulation and inhibitory process dysfunctions
Sundaresan et al. (20)	EEG	16 channels (Fp1, Fp2, C3, C4, P7, P8, O1, O2, F7, F8, F3, F4, T7, T8, P3, P4).	Stress-related EEG classification using LSTM; achieved 93.27% accuracy	Feasibility of EEG-based BCI for stress assessment in ASD
Mason et al. (21)	EEG	62 channel montage, referenced to FCz.	Delayed N170 latency in response to faces; related to fusiform activity	Biomarker for stratifying ASD subgroups and predicting social prognosis
Busra et al. (22)	EEG	21 channels (P3, C3, F3, Fz, F4, C4, P4, Cz, A1, Fp1, Fp2, T3, T5, O1, O2, F7, F8, A2, T6, and T4)	Decreased beta synchronization between frontal and temporal regions	Impaired inter-regional communication for social/sensory processing
Rogala et al. (23)	EEG	18 channels in the 10–20 system.	Reduced beta synchrony; altered alpha/beta connectivity	Disrupted global and inter-regional brain communication in ASD
Xu et al. (24)	fNIRS	44 channels, 22 in each hemisphere, on inferior frontal gyrus (1–10, 23–32) and temporal lobe (11–22, 33–44).	Non-stationary temporal dynamics in optical channels	Potential biomarkers for ASD classification
Zhu et al. (25)	fNIRS	44 channels, 22 in each hemisphere, on inferior frontal gyrus (1–10, 23–32) and temporal lobe (11–22, 33–44).	Enhanced connectivity in LIFG	Disruption in language-related neural circuits
Xu et al. (26)	fNIRS	44 channels, 22 in each hemisphere, on inferior frontal gyrus (1–10, 23–32) and temporal lobe (11–22, 33–44).	Deep network (CNN-LSTM-Attention) identified key signals in LTL	High diagnostic accuracy; importance of LTL in ASD
Xu et al. (27)	fNIRS	44 channels, 22 in each hemisphere, on inferior frontal gyrus (1–10, 23–32) and temporal lobe (11–22, 33–44).	Lower SampEn in IFG and TC, particularly in left hemisphere	Abnormal self-similarity in ASD brain activity; supports fNIRS-based diagnosis
Xu et al. (28)	fNIRS	44 channels, 22 in each hemisphere, on inferior frontal gyrus (1–10, 23–32) and temporal lobe (11–22, 33–44).	Lower fluctuation entropy in ASD from a single fNIRS channel; achieved 97.8% classification accuracy	High self-similarity in ASD spontaneous brain activity; supports single-channel fNIRS as a biomarker
Gui et al. (29)	EEG	128 channels in the 10–20 system	Differences in Nc ERP and attentive microstate strength in infants with family history of ASD	Early attention engagement deficits may predict ASD and social skill outcomes

### 3.2 Signal processing techniques

Preprocessing techniques for EEG signals are crucial to improving data quality by removing artifacts, particularly those caused by muscle and eye movements. Dickinson et al. proposed a systematic pipeline beginning with high-pass and low-pass filtering to exclude frequencies below 1 Hz and above 90 Hz, followed by visual inspection to identify evident artifacts such as electromyographic activity. More subtle artifacts are addressed using Artifact Subspace Reconstruction (ASR) with Principal Component Analysis (PCA) to isolate high-amplitude disruptions. Independent Component Analysis (ICA) is then applied to separate brain signals from ocular, muscular, and cardiac sources, significantly enhancing signal quality (30). Similarly, Imah et al. employed PCA after z-score normalization to reduce inter-subject variability, combined with Discrete Wavelet Transform (DWT) for noise reduction, effectively decreasing computational complexity while maintaining accuracy (31). Melinda et al. demonstrated the effectiveness of combining ICA with DWT, particularly using the Daubechies 4 wavelet to target specific frequencies, yielding optimal noise reduction (32). On the other hand, Jayanthy used Continuous Wavelet Transform (CWT) with Morlet wavelets for feature extraction, preserving discriminative information. However, this method’s computational intensity makes it less practical for real-time applications (33).

Manual preprocessing approaches, like those of Garcés et al., apply band-pass filtering (1–32 Hz) and manually eliminate segments with artifacts, followed by ICA for muscular artifact removal. This approach is effective but time-intensive for complex datasets (34). Sundaresan et al. employed a similar band-pass filter in real-time Brain-Computer Interface applications, offering faster preprocessing but potentially lower accuracy (20). Automated systems have also gained traction. Abou-Abbas et al. used a system dividing signals into non-overlapping 1-second windows, calculating the standard deviation to identify artifacts (35). Han et al. leveraged EEGLAB software to downsample data to 250 Hz, applied a 50 Hz notch filter for power line noise, and implemented a band-pass filter (0.5–45 Hz) to mitigate aliasing (36). Finally, Bouallegue et al. present a novel strategy that incorporates both FIR (Finite Impulse Response) and IIR (Infinite Impulse Response) filters where the first, are chosen for their ability to preserve the linear phase and minimize approximation error while the latter, are selected for their computational efficiency, providing near-ideal responses with fewer coefficients, thereby reducing computational complexity (37).

In contrast, fNIRS signals require less preprocessing, addressing primarily physiological and motion-induced noise. Li et al. applied a first-order polynomial filter for signal drift removal and a wavelet-based approach to separate relevant components from motion artifacts. Physiological noise was minimized using a second-order Butterworth filter (0.009–0.08 Hz), followed by z-score normalization for channel standardization. Data augmentation using Gaussian noise expanded the dataset fivefold, enhancing model reliability (38). Xu et al. further tackled sample size limitations with a moving window technique, segmenting temporal data into overlapping subsequences. This method enriched the dataset, improving classification robustness and enabling more precise analysis (39). The main findings of the studies reported in this section are summarized in **Table 3**.

**Table 3:** Summary of EEG and fNIRS preprocessing techniques, including filtering strategies, artifact correction methods, and data augmentation approaches used to enhance signal quality and model performance.

Authors	Signal type	Preprocessing techniques	Benefits
Dickinson et al. (30)	EEG	High-pass/low-pass (1–90 Hz), visual inspection, ASR with PCA, ICA	High signal quality, effective removal of subtle and overt artifacts
Imah et al. (31)	EEG	Z-score normalization, PCA, DWT	Reduced variability, efficient noise reduction, manageable complexity
Melinda et al. (32)	EEG	ICA + DWT (Daubechies 4)	Targeted frequency noise removal, optimized signal quality
Jayanthy (33)	EEG	CWT with Morlet wavelets	Preserved discriminative features, not ideal for real-time use
Garcés et al. (34)	EEG	Band-pass (1–32 Hz), manual artifact elimination, ICA	Effective, labor-intensive for complex datasets
Sundaresan et al. (20)	EEG	Band-pass filtering	Fast preprocessing for real-time BCI, reduced accuracy
Abou-Abbas et al. (35)	EEG	Segmentation into 1s windows, standard deviation for artifact detection	Automated, scalable artifact identification
Han et al. (36)	EEG	Downsampling to 250 Hz, 50 Hz notch, band-pass (0.5–45 Hz)	Filtered aliasing and power line noise, good preprocessing quality
Bouallegue et al. (37)	EEG	FIR and IIR filters	Balanced phase fidelity and computational efficiency
Li et al. (38)	fNIRS	Polynomial filtering, wavelet, Butterworth (0.009–0.08 Hz), z-score, Gaussian noise	Controlled motion/physiological noise, boosted model robustness
Xu et al. (39)	fNIRS	Moving window segmentation	Sample size enrichment, improved classification precision

### 3.3 Classification algorithms and models employed

Algorithms of ML and DL have been found to be of great aid in the early diagnosis of ASD, thanks to their intrinsic ability to learn from data and make decision based on the patterns observed. The output of said models, in most cases, is a metric of accuracy, indicating how many instances have been correctly classified.

Leveraging EEG signals, Abou-Abbas et al. employed standard ML algorithms, such as Support Vector Machine (SVM) and k-Nearest Neighbors (kNN) to classify familial risk of ASD development, reaching an accuracy of 88.44% with the former model (35). The robustness of SVM was further corroborated in studies like that by Aslam et al., which reported a median accuracy of 98.60% across multiple EEG datasets for emotional assessment and ASD diagnosis (40). Similarly, Martinez and Chen compared SVM with advanced models like LASSO Logistic Regression (LR) and Random Forest (RF) to analyze sleep patterns in ASD, obtaining accuracies of 87.00%, 83.00%, and 85.00%, respectively (19). In contrast, Imah et al. demonstrated the superiority of a more advanced ML algorithm, Generalized Relevance Learning Vector Quantization, achieving an accuracy of 73.28% in analyzing EEG channels related to joint attention (31). Finally, Melinda et al. highlighted the potential of Linear Discriminant Analysis (LDA), which delivered a remarkable accuracy of 99.00% (32).

DL models have consistently outperformed traditional ML approaches in classifying EEG signals for ASD. Jayanthy compared SVM with architectures like Convolutional Neural Networks (CNNs) and Long Short-Term Memory Networks (LSTMs), achieving a top accuracy of 94.00% with CNNs (33). Tawhid et al. extended this comparison, using spectrogram images of EEG signals, and reported maximum accuracies (41). Bouallegue et al. further enhanced CNN performance by integrating advanced filtering techniques, reaching an accuracy of 99.50% in detecting ASD-specific patterns (37). Among advanced DL methodologies, Radhakrishnan et al. tested popular architectures such as ResNet50, DenseNet, and AlexNet for automatic ASD detection, achieving their best accuracy, 81.91%, with ResNet50 (42). Han et al. proposed a more sophisticated approach, combining multimodal feature learning and a stacked denoising autoencoder, which yielded an accuracy of 98.40% (36).

Of particular interest is the research proposed by Sundaresan et al. in which advanced DL models were applied to classify stress states in adolescents with ASD and neurotypical peers. Models evaluated included SVM with Filter Bank Common Spatial Patterns (FBCSP), CNNs, such as Deep ConvNet and Shallow ConvNet, and LSTMNs with one, two, or three layers. Among these, the two-layer LSTM network exhibited the highest classification performance, achieving an accuracy of 93.27% in distinguishing stress states (20).

In the context of fNIRS data, the complexity of the signals has led to a predominance of DL models. However, traditional ML approaches have also demonstrated their effectiveness in specific scenarios. Xu et al. compared several ML algorithms, including SVM, Gaussian Naïve Bayes, Logistic Regression, Multi-Layer Perceptron, Decision Tree, and Network Cluster, with the latter achieving the highest accuracy of 97.80% (28). Zhang et al. reaffirmed the robustness of SVM, achieving an accuracy of 88.00% in classifying Autism Diagnostic Observation Schedule (ADOS) scores (43). Additionally, Xu et al. employed a k-means clustering algorithm for distinguishing ASD patients from TD subjects using data from the temporal cortex, achieving an accuracy of 97.60% (27).

Advanced DL algorithms, particularly CNNs and LSTMs, have been widely adopted. Li et al. introduced a dual-branch DL architecture combining CNNs and LSTMs with an attention mechanism, achieving an accuracy of 94.00% in ASD detection (38). Shin et al. obtained the same accuracy using a Bidirectional LSTM coupled with a CNN to distinguish between ADHD and ASD (44). LSTMs alone also proved effective in Xu et al. study, achieving an accuracy of 93.30% in classifying signals from the left temporal lobe of ASD and TD individuals (26).

More advanced CNN-based models have yielded optimal results in recent studies. For instance, Adaptive Graph Neural Networks, applied to fNIRS signals from the left inferior frontal gyrus, achieved an accuracy of 97.10%, outperforming LSTM models (25). Xu et al. demonstrated that combining CNNs with Gated Recurrent Units to extract both spatial and temporal features from fNIRS data resulted in an accuracy of 92.20% for ASD and TD classification (39). Moreover, the integration of an Adaptive Spatio-Temporal Graph Convolution Network further improved performance, achieving an accuracy of 95.40% (45). The findings reported in this section are reported in **Table 4**.

**Table 4:** Classification performance of machine learning and deep learning algorithms applied to EEG and fNIRS data for ASD diagnosis, including model types, input modalities, and reported accuracies.

Authors	Numbers of patients and age range	Features employed	ML or DL model	Cross-validation	Results
Abou-Abbas et al. (35)	104 infants, (included 94, 35 males and 59 females)This sample was divided into 17 high-risk infants who received a diagnosis (HR-ASD), 33 high-risk infants who did not receive a diagnosis (HR-noASD), and 44 control infants.	Energy, Shannon entropy, standard deviation, skewness, moment, and mean of EEG signals processed with empirical mode decomposition.	SVM, kNN	Training and testing using nested cross validation: 9 ×10 (number of inner folds _ number of outer folds) for the first classification problem (HR vs. control). A 4 ×5 cross validation was used in the second classification problem (HR-ASD and HR-noASD)	Maximum Accuracy of 88.44% attained with SVM
Aslam et al. (40)	99 subjects: 70 and 29 subjects for emotions and ASD classification, respectively.	A selection of the following based on the specific EEG dataset employed in the study: Fast Fourier transform coefficients, skewness, mean, signal energy, continuous wavelet transform coefficients, auto-correlation, absolute energy, mean absolute change, mean second derivative, change quantiles, energy ratio, and linear trend	LSVM, DNN, KNN,DT, and XGB classifiers	Different datasets are employed and chosen as validation set.	Median Accuracy across all dataset: 98.60%
Martinez and Chen (19)	149 ASD patients and 197 age-matched controls from the Nationwide Children’s Health (NCH) dataset, and an additional 79 age-matched controls from the Childhood Adenotonsillectomy Trial (CHAT) dataset. Furthermore, a smaller independent NCH cohort included 38 autism patients and 75 controls aged 0.5–3 years for further validation.	Four macro-group of features derived from EEG signals of sleeping stages, comprising Polysomnogram, Spectral Features, Spindle/Slow Wave Features and Aperiodic Features	SVM, LASSO LR and RF	10-fold stratified cross-validation with 20 repeats. Moreover, an external validation using an independent pediatric sleep study (CHAT) was implemented.	Maximum Accuracy of 87.00% attained with SVM
Imah et al. (31)	The dataset utilized in the study is the BCIAUT-P-300. It comprises EEG signals from 15 ASD subjects, aged between 16 to 38 years, all male, diagnosed based on the Autism Diagnostic Interview-Revised, Autism Diagnostic Observation Schedule, and the DSM-5 criteria.	Wavelet coefficients extracted from EEG signals	RF, Backpropagation, SVM and Generalized Relevance Learning Vector Quantization	The dataset consists of train data and test data for each subject with 7 sessions.	Maximum Accuracy of 73.28% attained with Generalized Relevance Learning Vector Quantization
Melinda et al. (32)	16 children, with an equal division between eight autistic and eight tipically developing individuals.	DWT coefficients extracted from EEG signals	Linear Discriminant Analysis (LDA)	70% training process and 30% testing	99.00% Accuracy
Mohi ud Din & Jayanthy (33)	28 subjects divided in 14 ASD children and 14 healthy controls. Additionally, another dataset including13 ASD and 4 healthy controls.	Wavelet Scattering Transform coefficient extracted from EEG signals	SVM, CNN, LSTM	80:20 for train and test dataset	Maximum Accuracy of 94.00% attained with CNN
Tawhid et al. (41)	16 subjects: 12 with ASD (3 girls and 9 boys, age 6–20 years of age) and 4 healthy subjects (all boys, 9–13 years of age).	Ternary CENTRIST features extracted from spectrograms of EEG signals	CNN	The dataset is divided into 70%, 15%, 15% for training, validation and testing; 10-fold cross-validation	99.15% Accuracy
Bouallegue et al. (37)	10 healthy volunteers and 9 autistic subjects (6 males and 3 females) who are aged from 6 to 16 years	ICA feature extracted from EEG signals	CNN	10-fold cross-validation	99.50% Accuracy
Radhakrishnan et al. (42)	20 participants, comprising 10 typically developing children and 10 ASD children.	EEG spectrograms plotted through a Discret Fourier Transform method	ResNet50, DenseNet, AlexNet	The dataset was split into two nonoverlapping sets: 80% for training and 20% for testing; 5-fold cross-validation	Maximum Accuracy of 81.91% attained with Resnet50
Han et al. (36)	90 subjects:40 ASD and 50 typically developing children aged between 3 to 6 years.	Relative Power Energy, Multiscale Entropy and Brain Function Network from EEG signals, combined with specific features from Eye Tracking data	Stacked Denoising AutoencoderA two-step multimodal feature learning and fusion model, multimodal stacked denoising autoencoder (MMSDAE) consisting of two core modules: unimodal feature learning module and multimodal feature fusion module.	10-fold subject-independent cross-validation	98.40% Accuracy
Sundaresan et al. (20)	13 participants, with 8 ASD and 5 neurotypical adolescents.	Processed EEG signals	SVM with FBCSP, CNN (Deep ConvNet, Shallow ConvNet), LSTM	80:20 for train and test dataset	Maximum Accuracy of 93.27% attained with Two-Layer LSTM
Xu et al. (28)	25 ASD children (age 9.3 ± 1.4 years) and 22 age-matched typically developing children	Fluctuation Entropy distribution across fNIRS channels	SVM, Gaussian NB, LR, MLP, Decision Tree, Network Cluster	10-fold cross-validation. Finally, the test set is put into the network for ASD discrimination.	Maximum Accuracy of 97.80% attained with Network Cluster
Zhang et al. (43)	17 ASD adults (3 female; mean age 25 ± 4.9 years; 12 right-handed, 3 left-handed, and 2 ambidextrous) whose diagnoses were verified by research-reliable clinician assessments, including the Autism Diagnostic Observation Schedule, 2nd Edition (ADOS-238). 19 typically developed adults (mean age 26 ± 5.8 years; 18 right-handed and 1 ambidextrous) also participated. Groups were matched by age, gender, and IQ.	Spatial Features of fNIRS signals analyzed with PCA	SVM	Nested-cross validation	88.00% Accuracy
Xu et al. (27)	25 ASD children (age 9.3 ± 1.4 years) and 22 age-matched typically developing children	Sample Entropy distribution across fNIRS channels	k-Means Clustering	60% of the participants are selected as training set and the remaining 40% are selected as test set.	97.60% Accuracy
Li et al. (38)	25 children with ASD and 22 children with TD.	Multiscale Entropy of across fNIRS channels	CNN + LSTM	4-fold cross-validation	94.00% Accuracy
Shin et al. (44)	13 ADHD children with coexisting ASD and 15 typically developing children	Processed fNIRS signals	Bidirectional LSTM + CNN	Leave-One-Out Cross Validation (LOOCV)	94.00% Accuracy
Xu et al. (26)	25 children with ASD and 22 age-matched typically developing children	Local fNIRS signal features extracted with a CNN algorithm	LSTM	The training set, verification set and test set were divided according to the proportion of 6:2:2. The classes were balanced.	93.30% Accuracy
Zhu et al. (25)	25 children with ASD (age 9.3 ± 1.4 years) and 22 age-matched typically developing children (9.5 years ±1.6)	Spatio-temporal fNIRS signal features extracted through graph neural network combined with the temporal convolution module	Adaptive Graph Neural Networks	Leave-One-Out Cross Validation (LOOCV)	97.10% Accuracy
Xu et al. (39)	25 children with ASD (age 9.3 ± 1.4 years) and 22 age-matched typically developing children (9.5 years ±1.6)	Processed fNIRS signals	CNN + GRU	Dataset divided in training set, validation set, and test set. Their proportion is 3:1:1.	92.20% Accuracy
Zhang et al. (45)	25 children with ASD (age 9.3 ± 1.4 years) and 22 age-matched typically developing children (9.5 years ±1.6)	Spatio-temporal fNIRS signal features extracted through graph neural network	Adaptive Spatio-Temporal Graph Convolution Network	The training set, verification set and test set were divided according to the proportion of 6:2:2. The classes were balanced.	95.40% Accuracy

### 3.4 Inter-study agreement analysis

The inter-study agreement analysis highlights a substantial heterogeneity inherent in the reviewed literature. Regarding neurophysiological findings, no single biomarker achieved a prevalence above 33.3%, as shown in **Table 5**. Similarly, regarding the preprocessing strategies, a lack of standardized pipelines is shown. In fact, widely used techniques such as ICA and band-pass filtering were explicitly reported in only 27.3% of the studies, as reported in **Table 6**.

**Table 5:** Inter-study agreement analysis for neurophysiological findings.

Variable	Count (k/N)	Prevalence (%)	Agreement	PABAK	Fleiss’ Kappa (κ)	Krippendorff’s α
Theta-band alteration	2/12	16.7	0.697	0.394	-0.09	-0.09
Alpha-band alteration	2/12	16.7	0.697	0.394	-0.09	-0.09
Beta-band alteration	2/12	16.7	0.697	0.394	-0.09	-0.09
Connectivity/synchrony alteration	3/12	25.0	0.591	0.182	-0.12	-0.11
Frontal region involvement	3/12	25.0	0.591	0.182	-0.12	-0.11
Temporal region involvement	4/12	33.3	0.515	0.030	-0.11	-0.10
Inferior frontal gyrus involvement (IFG/LIFG)	2/12	16.7	0.697	0.394	-0.09	-0.09
Left temporal lobe involvement (LTL)	1/12	8.3	0.833	0.667	-0.05	-0.05
Fusiform gyrus involvement	1/12	8.3	0.833	0.667	-0.05	-0.05
ERP/microstate marker (N170/Nc)	2/12	16.7	0.697	0.394	-0.09	-0.09
Entropy-based metric (SampEn/Fluctuation Entropy)	2/12	16.7	0.697	0.394	-0.09	-0.09
Non-stationary hemodynamic dynamics	1/12	8.3	0.833	0.667	-0.09	-0.09
Stress-related paradigm	1/12	8.3	0.833	0.667	-0.09	-0.09

**Table 6:** Inter-study agreement analysis for preprocessing strategies.

Variable	Conunt (k/N)	Prevalence (%)	Agreement	PABAK	Fleiss’ Kappa (κ)	Krippendorff’s α
High-pass/low-pass filtering	1/11	9.1	0.818	0.636	-0.10	-0.10
Band-pass filtering	3/11	27.3	0.564	0.127	-0.10	-0.10
Notch filtering (50 Hz)	1/11	9.1	0.818	0.636	-0.10	-0.10
Downsampling	1/11	9.1	0.818	0.636	-0.10	-0.10
Visual inspection	1/11	9.1	0.818	0.636	-0.10	-0.10
Manual artifact elimination	1/11	9.1	0.818	0.636	-0.10	-0.10
Artifact Subspace Reconstruction (ASR)	1/11	9.1	0.818	0.636	-0.10	-0.10
Principal Component Analysis (PCA)	2/11	18.2	0.673	0.345	-0.10	-0.10
Independent Component Analysis (ICA)	3/11	27.3	0.564	0.127	-0.10	-0.10
Discrete Wavelet Transform (DWT)	2/11	18.2	0.673	0.345	-0.10	-0.10
Continuous Wavelet Transform (CWT)	1/11	9.1	0.818	0.636	-0.10	-0.10
Finite Impulse Response (FIR) filters	1/11	9.1	0.818	0.636	-0.10	-0.10
Infinite Impulse Response (IIR) filters	1/11	9.1	0.818	0.636	-0.10	-0.10
Polynomial filtering/detrending	1/11	9.1	0.818	0.636	-0.10	-0.10
Wavelet-based denoising/filtering (unspecified)	1/11	9.1	0.818	0.636	-0.10	-0.10
Butterworth filtering	1/11	9.1	0.818	0.636	-0.10	-0.10
Z-score normalization	2/11	18.2	0.673	0.345	-0.10	-0.10
Gaussian noise augmentation	1/11	9.1	0.818	0.636	-0.10	-0.10
Windowing/segmentation (fixed or moving)	2/11	18.2	0.673	0.345	-0.10	-0.10
Std-based artifact detection	1/11	9.1	0.818	0.636	-0.10	-0.10

## 4 Discussion

### 4.1 Description of the results

This systematic review provides a comprehensive overview of current research regarding EEG and fNIRS combined with ML and DL methods applied for ASD identification. The search strategy allowed to select 27 studies that reveal the significant potential of this approach in capturing the complex neural signatures associated with ASD.

A key finding across EEG studies is the recurrent identification of abnormalities in specific frequency bands, particularly increased theta activity and disrupted alpha and beta band synchrony, predominantly in frontal and temporal regions. These findings support the hypothesis that ASD is characterized by impaired neural connectivity and atypical information processing, especially in circuits involved in attention, sensory integration, and social cognition (46–48). Similarly, fNIRS studies highlight alterations in hemodynamic responses in language and social brain regions, such as the inferior frontal gyrus and left temporal lobe. These results are aligned with clinical observations of language delays and social interaction difficulties in ASD, reinforcing the neurobiological underpinnings of the disorder (49).

From a methodological perspective, the review highlights significant advancements in signal preprocessing. For EEG, artifact removal techniques such as ICA, CWT, and advanced filtering strategies (FIR/IIR) have contributed to higher signal quality, a critical requirement for maximizing the ML and DL performance. While fNIRS preprocessing is generally less complex, effective procedure for filtering, motion artefact correction, and physiological contaminations removal can improve the signal quality and robustness.

Notably, several ML and DL models were investigated across the included studies, showing a notable effectiveness of artificial intelligence approaches to detect ASD derived patterns in EEG and fNIRS signals. While traditional ML methods like SVM remain popular due to their interpretability and robustness, it is worth to highlight that DL architectures, such as CNNs, LSTM networks, and GNNs, have shown superior performance in modeling complex temporal and spatial dynamics of brain activity. The highest reported classification accuracies, often exceeding 95%, underscore the efficacy of these approaches in distinguishing ASD from TD controls.

### 4.2 Comparative examination of EEG and fNIRS biomarkers across research studies

The examined literature highlights several neurophysiological abnormalities identified using EEG and fNIRS, supporting the implementation of a multimodal approach for the ASD-associated brain dysfunction examination.

EEG findings consistently reveal increased theta activity and decreased alpha/beta synchronization in frontal and temporal areas, reflecting an impaired development of fronto-temporal systems involved in executive functioning, attention regulation, or social perception. These electrophysiological findings are consistent with fNIRS evidence of altered patterns of hemodynamics in language- and social-associated areas (inferior frontal gyrus, superior temporal gyrus, left temporal lobe). Moreover, entropy-related metrics computed on both EEG and fNIRS reflect reduced temporal complexity and increased self-similarity in ASD-related brain activity. The cross-modal convergence of these markers supports the proposal that ASD is characterized by defective integration between localized neural activation and large-scale network coordination. Nevertheless, it is worth noting that some inconsistencies among the investigated studies have been reported (e.g., variable alpha asymmetry or lack of lateralization), but they could be ascribed to differences in age groups, task paradigms, and preprocessing pipelines across the studies considered.

Another aspect that should be investigated concerns the EEG and fNIRS fusion strategies. In fact, a complementary EEG and fNIRS features have been merged to improve the ASD identification, but approaches based on the fusion and interdependency of the two signals (e.g., conditional entropy between the two modalities, convolution between signals) should be more extensively explored.

### 4.3 Neurovascular coupling and connectivity disruptions

Studies employing simultaneous EEG–fNIRS demonstrate the dissociation of electrical activity from changes in hemodynamics, indicating impaired synchronization of neuronal firing and vascular regulation. This disruption may induce issues with the balance between excitation and inhibition, the development of cortical microcircuits, and long-range connectivity. Hence, the investigation of the studies reported in this review suggest that ASD involves modifications in local circuit dynamics and large-scale network organization, which can be accurately measured using combined EEG–fNIRS.

### 4.4 Divergence of DL models employed

From the reviewed literature, it emerges that DL models applied on EEG and fNIRS signals to identify ASD patients differ widely in architecture. For instance, CNN-based models are often employed on EEG signals (e.g., DeepConvNet, ShallowConvNet, ResNet variants), extracting spatial–temporal representations of oscillatory activity. Regarding the fNIRS, RNN-based models (LSTM, GRU, Bi-LSTM) are commonly employed to investigated the hemodynamic patterns.

Hybrid architectures, such as CNN–LSTM or graph-based neural networks (GNNs), are increasingly applied on both fNIRS and EEG, yielding to high accuracies when detecting ASD.

### 4.5 The effect of preprocessing pipelines on ML/DL models performance

An in-depth study of preprocessing techniques shows that the diversity of the algorithms has a direct impact on the signal quality and feature reliability when it comes to model interpretation.

EEG studies with ICA, ASR, or wavelet denoising show that the classifier performance is consistently improved across methods, indicating that a successful cleanup artifact procedure is required to identify subtle ASD-related patterns. In contrast, pipelines involving only basic band-pass filtering are less accurate and quite variable, particularly for DL models that are more sensitive to noise.

For fNIRS, approaches that integrate motion-artifact correction (e.g., wavelet filtering and PCA-based regressors) with removal of low-frequency physiological noise result in more consistent hemodynamic signals, as well as improved classifier performance.

These findings confirm that harmonizing the processing pipeline is important for reproducibility and endorse the perception that differences in published accuracies are predominantly due to methodological inconsistencies.

### 4.6 The effect of cross-validation in ML/DL performance

Three methodological trends can be observed across the studies that were reviewed. First, conventional ML methods (e.g., SVM, LDA, and LR) are effective only if the extracted features are selected well. Nevertheless, DL architectures are more successful using properly processed signals as inputs since they exploit the temporal and spatial structure. Second, many studies with small sample sizes may employ data augmentation or windowing strategies, which add more samples without increasing diversity among subjects. Third, regarding cross-validation, some studies employ a subject-dependent or non-stratified cross-validation, which increases the chance of data leakage and overestimating the accuracy. A more rigorous standardization of validation procedures is necessary to enable direct comparison across studies and assess genuine model generalizability.

In detail, in several of the investigated works, validation was performed using simple random splits (e.g., 70/30 or 80/20) or k-fold procedures applied directly to the samples rather than at the subject level. Notably, this approach introduces a risk of data leakage since trials from the same participant may be present in both the training and test folds, thus inflating classification performance. Additionally, several studies applied k-fold or LOOCV strategies to small study samples, leading to high-variance performance estimates. Only few studies implemented subject-independent or nested cross-validation, which are suitable to reduce overfitting. However, it should be noted that the heterogeneity and instability of the cross-validation approaches used limit the comparability of the results. Finally, many studies employed in-house datasets, limiting reproducibility and preventing independent verification of the reported results.

Although considering the previous mentioned methodological limitations, the mean accuracy across the studies was 93.39% with a standard deviation of 6.50% and a coefficient of variation of 6.9%, suggesting that EEG- and fNIRS-based ML/DL approaches can achieve high discriminative performance for ASD.

### 4.7 The effect of the ASD identification criteria and sample size on ML/DL performance

Another crucial aspect that should be examined is the heterogeneity in the diagnostic and screening tools employed for ASD identification. In fact, while some studies relied on ICD-10 clinical codes (F84.0) extracted from electronic medical records, others adopted standardized behavioral assessments such as the Autism Diagnostic Observation Schedule (ADOS) or the Autism Diagnostic Interview–Revised (ADI-R) to confirm the diagnosis. It should be fostered the adoption of standardized multi-level diagnostic frameworks (e.g., combining ADOS/ADI-R behavioral profiles with clinical data) to ensure that ML models trained on neuroimaging features can generalize across populations and developmental stages. This approach will allow for the development of models that not only identify the pathology, but also predict the symptoms progression.

Finally, it should be highlighted that some of the examined studies have a reduced number of participants (below 30 participants per class), thus limiting the generalizability of the findings. In fact, although the algorithms are cross-validated, an increased study sample could improve the performance of the models and the generalizability of the results.

### 4.8 Clinical translation

The robustness of the findings reported in the investigated papers foster the development of recommendations for translating these insights into clinical practice. In fact, early screening in infants could employ portable EEG/fNIRS to detect atypical development as young as 6–12 months, enabling proactive intervention. Diagnostic evaluations for toddlers and children can be bolstered by brief EEG/fNIRS exams, support clinical diagnoses by adding objective physiological evidence related to both cerebral electrical activity, hemodynamics, and neurovascular coupling. Moreover, the periodical monitoring of brain activities modification over time can support the treatment monitoring, confirming the effectiveness of the therapy. However, the clinical translation of EEG/fNIRS-based ML and DL tools remains limited due to several practical factors, such as equipment cost, portability, required acquisition time, and the availability of trained personnel. Hence, standardized protocols, cost-effective hardware solutions, and user-friendly interfaces will be essential for transitioning these approaches from research environments to routine clinical practice.

### 4.9 Montage divergence across studies

Another crucial aspect that should be examined regards the variability among EEG and fNIRS montages. Specifically, EEG montages range from low-density (8–16 channels) to high-density (64–128 channels), whereas fNIRS montages vary even more widely, targeting IFG, STG, temporal cortex, and prefrontal regions, with a limited number of channels.

Heterogeneous montages limit direct comparison of spatial findings and introduce variability in DL models trained on different sensor configurations, hence standardized whole-head configurations should be fostered, in order to endorse multicentric data-exchange and the development of consistent models.

### 4.10 Inter-study agreement

The inter-study agreement analysis demonstrated a strong heterogeneity regarding both neurophysiological findings (**Table 5**) and preprocessing strategies (**Table 6**). These results underscore in a quantitative manner the methodological variability described qualitatively in the review, demonstrating that current research protocols are too diverse to support a direct statistical meta-analysis. Crucially, the chance-corrected Krippendorff’s α values were consistently low or negative (ranging from -0.05 to -0.12) across all variables, including common techniques like ICA or Band-pass filtering. These near-zero coefficients statistically confirm the urgent need for standardized protocols in this research domain.

### 4.11 Limitations and future directions

Several challenges persist in this research field. For instance, the majority of studies rely on small, often non-standardized datasets, limiting generalizability and external validation. Moreover, the integration of EEG and fNIRS remains scarce, despite the theoretical and practical advantages of multimodal approaches. In fact, the employment of multimodal EEG-fNIRS allows detecting a disrupted neurovascular coupling, that this the functional hyperemia accompanying the neurons’ activation, and providing concurrent information on both electrical and hemodynamic brain activity (50, 51). Future research should indeed prioritize the development of larger, harmonized datasets that combine EEG and fNIRS data, potentially through multi-center collaborations.

Another limitation regards to the heterogeneity among the included studies regarding sample size, age ranges, experimental paradigms, preprocessing pipelines, and validation strategies. This variability makes it harder to compare results and limits the generalizability of the findings. Another crucial limitation is related to the limited interpretability of DL models, which function as “black boxes” and offer limited insights into underlying neurobiological mechanisms. Importantly, Explainable Artificial Intelligence (XAI) frameworks are designed to provide insights into how the model reaches its predictions, thus enabling stakeholders to better understand and trust the DL models’ outputs. A significant advantage of XAI relies on its capability to enhance model robustness. Through explainability, developers can better understand failure points within their models and make informed adjustments to improve performance. This iterative process, driven by understanding model behaviors and the contextual applicability of predictions leads to more reliable AI systems that can adapt to varying conditions and requirements. Importantly, XAI strengthens collaborative interactions between human experts and AI systems. In fact, by elucidating how decisions are made, XAI facilitates the inclusion of human judgment in the decision-making process, leading to more contextualized and validated outcomes. This advantage is particularly relevant in medicine, where diagnostic decisions derived from AI recommendations must integrate human expertise of physicians for successful implementation (52).

Finally, further studies should be performed investigating the feasibility to apply this approach in telemedicine and Internet of Things (IoT) contexts, including the development of portable, user-friendly systems capable of performing reliable early screening in clinical or home settings.

## 5 Conclusions

This review highlights the growing potential of neuroimaging techniques, particularly EEG and fNIRS, combined with ML/DL methods, for early diagnosis of ASD. EEG and fNIRS offer complementary insights into the neurophysiological alterations characteristic of ASD, leveraging EEG’s exceptional temporal resolution and the fNIRS ability to assess hemodynamic changes. Through the integration of advanced ML and deep learning DL algorithms, researchers have achieved promising accuracy levels in distinguishing individuals with ASD from typically developing controls. Key neurophysiological biomarkers, such as abnormalities in EEG frequency bands and disrupted connectivity in brain regions associated with social cognition and language processing, have been consistently reported. Similarly, fNIRS studies have identified hemodynamic alterations in areas like the inferior frontal gyrus and superior temporal gyrus, further supporting the role of these regions in ASD. Preprocessing pipelines have been pivotal in ensuring signal quality, with methods like ICA for EEG and wavelet-based filtering for fNIRS emerging as gold standards. The adoption of ML and DL algorithms has significantly advanced the field, with models such as SVM, CNNs, LSTMs demonstrating high classification performance. Despite their computational demands, these algorithms have proven effective in handling the complexity of neuroimaging data and enhancing diagnostic precision.

Concluding, combining neuroimaging techniques with advanced computational approaches offers significant potential to revolutionize the diagnosis of ASD. By closing the divide between scientific research and practical clinical use, these advancements could greatly enhance early detection and intervention methods, leading to better outcomes and quality of life for individuals with ASD.

## Data Availability

The original contributions presented in the study are included in the article/supplementary material. Further inquiries can be directed to the corresponding author.
